# Nonconforming gender expression and adolescent anabolic-androgenic steroids misuse

**DOI:** 10.1186/s13034-024-00761-9

**Published:** 2024-06-06

**Authors:** Ruili Li, Yuexi Liu, Qiguo Lian

**Affiliations:** 1 Capital Institute of Pediatrics, Beijing, China; 2 The Second Clinical Medical College, Dalian Medical University, Dalian, China; 3 NHC Key Lab of Reproduction Regulation, Shanghai Institute for Biomedical and Pharmaceutical Technologies, 779 Laohumin Road, Shanghai, 200237 China

**Keywords:** Anabolic-androgenic steroids, Gender nonconformity, Gender expression, Youth Risk Behavior Survey

## Abstract

**Background:**

Gender nonconformity (GNC) is an under-researched area of adolescent health that is of increasing interest to researchers and general public. However, little is known about whether it is associated with anabolic-androgenic steroids (AAS) misuse. We aimed to investigate the association among high school students using a cross-sectional design.

**Methods:**

We pooled the 6 school districts data from the Youth Risk Behavior Survey in 2017 and 2019. We compared the prevalence of AAS misuse among gender nonconforming and conforming students. AAS misuse was determined on the reported experience of lifetime non-prescription steroid use. GNC was derived from perceived gender expression and sex. We estimated the sex-stratified adjusted odds ratios (AORs) for the association of GNC with AAS misuse after adjusting for race/ethnicity, grade, and sexual orientation.

**Results:**

The study population consisted of 17,754 US high school students including 9143 (49.67%) female students. Among female students, GNC was significantly associated with moderate (AOR, 3.69; 95% CI 1.28–10.62; *P* = 0.016) and severe (AOR, 5.00; 95% CI 1.05–23.76; *P* = 0.043) AAS misuse, but not with any AAS misuse (AOR, 0.85; 95% CI 0.34–2.14; *P* = 0.734). Among male students, GNC was significantly associated with any (AOR, 4.75; 95% CI 2.93–7.69; *P* < 0.001), moderate (AOR, 4.86; 95% CI 2.66–8.89; *P* < 0.001) and severe (AOR, 4.13; 95% CI 1.43–11.95; *P* = 0.009) AAS misuse. We did not observe a dose-response relationship between GNC and any AAS misuse in female and male students.

**Conclusions:**

This study suggests that AAS misuse is associated with GNC among female and male adolescents.

**Supplementary Information:**

The online version contains supplementary material available at 10.1186/s13034-024-00761-9.

## Introduction

Gender expression (i.e., the way in which a person acts to present gender to others through physical appearance, clothing choice and accessories, and behaviors within a given culture [1]) can directly shape health behaviors and outcomes for human beings. Existing research suggests a link between masculinity and heavy episodic drinking in college-aged men [2], and a link between femininity and increased depression in young women [3]. Gender nonconformity (GNC) refers to gender expression differs from societal expectations prescribed for people of a particular sex [4]. Gender nonconforming adolescents are more likely to experience a variety of minority stressors [5, 6], and are more vulnerable to a range of problem behaviors, such as problematic smartphone use [7] and substance use [8].

Anabolic-androgenic steroids (AAS) misuse in adolescents is a growing public health concern. AAS are a family of hormones, including testosterone and synthetic derivatives, that facilitate muscle development and fat loss [9]. AAS misuse has a range of irreversible prolonged adverse effects on many organ systems, leading to not only medical and psychiatric pathology [10], but also emotional and behavior dysfunctions [11]. AAS misuse is not a relatively infrequent phenomenon among adolescent. In the United States as an example, the nationwide prevalence of self-reported lifetime misuse of steroids is 2.9% among high school students in 2017 [12]. In addition, research suggests roughly one-quarter of AAS misusers starts in adolescence [13]. Adolescents biologically are more vulnerable to the effects of AAS because of their developing brain and body, making them as the key group to study, even though they constitute a minority of AAS misusers [14].

Evidence suggests that AAS misuse among adolescent boys is higher than among girls (3.3% in boys and 2.4% in girls) [12], and sexual minority adolescent boys are more likely to report AAS misuse than their heterosexual counterparts [15]. The gender and sexual orientation disparities in AAS misuse can be partly attributed to gender expression. Generally, men are more probable to conform to traditional masculinity norms compared with women [16], sexual minorities (e.g., lesbian, gay, and bisexual individuals) tend to be more gender nonconforming than their heterosexual peers [8]. GNC is associated with elevated mental health disorders in adolescent boys and girls due to minority stress [8, 17], and depressive symptoms such as suicidal ideation are linked with AAS misuse [15].

To date, little is known about the association of GNC with AAS misuse during adolescence. While it is clear that sexual minority adolescent boys experience increased AAS misuse compared with heterosexual counterparts, the association of GNC with AAS misuse among the general population of adolescents independent of sexual orientation is worthy of further investigation. To bridge this gap, we extracted data from Youth Risk Behavior Survey (YRBS) to estimate the direction and strength of association between GNC and AAS misuse among US high school students.

## Methods

### Data source and sample

This cross-sectional study was based on an analysis of the pooled district YRBS data in 2017 and 2019, and YRBS data are publicly available from Youth Risk Behavior Surveillance System on the website of US Centers for Disease Control and Prevention (www.cdc.gov/healthyyouth/data/yrbs). In 2017, 20 large urban school districts conducted YRBS using a 2-stage cluster sample design to produce a representative sample of high school students, and the number of school districts increased to 29 in 2019. Because items on the YRBS pertaining to gender expression and AAS misuse are optional for school districts, only participants in 6 school districts received these items (Table 1). We combined 6 school districts from YRBS in 2017 and 2019 respectively into a single dataset to increase the sample size (*n* = 18,001). We dropped 247 cases that information on sex is missing, leaving a sample of 17,754. Overall response rates ranged between 61 and 85% in 2017 and between 61 and 86% in 2019 for the 6 selected school districts.

**Table 1:** Unweighted sample size by survey year, sex and location in selected US school districts, 2017–2019

Location	Total	Survey year		Sex	
		2017	2019	Female	Male
Broward County, FL(FT)	2099	931	1168	1190	909
Chicago, IL (CH)	3389	1851	1538	1732	1657
Los Angeles, CA (LO)	2717	1402	1315	1358	1359
Oakland, CA (OA)	2952	1947	1005	1506	1446
Philadelphia, PA (PH)	2780	1574	1206	1488	1292
San Diego, CA (SA)	3817	2442	1375	1869	1948

The district YRBS was reviewed and approved within each school districts using their local procedures. This study only used de-identified, publicly available YRBS data, and did not need further ethical approval.

### Measures

#### Exposure

We assessed the perceived gender expression with a validated self-reported question in YRBS [18]. Respondents were asked: “A person’s appearance, style, dress, or the way they walk or talk may affect how people describe them. How do you think other people at school would describe you?” with response options ranging on a seven-point scale from “very feminine” to “very masculine.” Response options followed with a 7-point scale: “1 = very feminine”, “2 = mostly feminine”, “3 = somewhat feminine”, “4 = equally feminine and masculine”, “5 = somewhat masculine”, “6 = mostly masculine,” and “7 = very masculine”.

We then created a 7-point GNC score measuring the degree of nonconformity to social norms of gender expression based on the self-reported perceived gender expression and sex. The GNC score labels the students from most gender conforming (1, indicating very feminine females and very masculine males) to most gender nonconforming (7, indicating very masculine females and very feminine males) [8]. We also generated a binary GNC variable, with 1 indicating gender conformity (GNC score: 1–4) and 2 indicating GNC (GNC score: 5–7) based on the result of preliminary data analysis.

### Outcomes

We assessed the AAS misuse with a self-reported question on lifetime non-prescription steroid use in YRBS. Respondents were asked: “During your life, how many times have you taken steroid pills or shots without a doctor’s prescription?” Response options followed with a 6-point scale: “1 = 0 times”, “2 = 1 or 2 times”, “3 = 3 to 9 times”, “4 = 10 to 19 times”, “5 = 20 to 39 times”, and “6 = 40 or more times”.

We then created a binary variable for any level of AAS misuse, with 0 indicating no misuse (Scale: 1) and 1 indicating all other responses (Scale: 2–6). We also generated two additional binary variables to assess moderate and severe levels of AAS misuse. Moderate misuse was defined as using AAS 10 or more times (Scale: 4–6), and severe misuse was defined as using AAS 40 or more times (Scale: 6). The three variables for different levels of AAS misuse were generated based on the existing literature on AAS misuse among adolescents [15, 19].

### Covariates

Based on existing evidence [5, 8, 19, 20], we identified potential confounders of the association between GNC and AAS misuse: sex (female or male), race/ethnicity (White, Black, Hispanic/Latino, or all other races), grade (9, 10, 11, or 12), and sexual orientation (heterosexual, lesbian/gay, bisexual, or unsure) [8, 12].

### Statistical analysis

The study had no prespecified statistical plan for analysis. We analyzed the data using a suite of *svy* commands in Stata/SE 15.1 (StataCorp LLC, College Station, TX, USA) to accommodate the complex sampling design (primary sampling units, stratum, weights) in YRBS [12]. We divided the weights by two to get the adjusted weights across years 2017 and 2019 for the combined data from district YRBS 2017 and 2019.

Prevalence estimates with 95% confidential intervals (CIs) was calculated using Taylor linearization approach [8]. Differences in the distributions of GNC and AAS misuse by covariates were examined using χ^2^ statistics. After adjusting for race/ethnicity, grade, and sexual orientation, the associations of GNC with three levels of AAS misuse were stratified by sex, because GNC and AAS misuse both varied by sex (Table S1). In initial analyses of association, we examined the linear association between GNC score and any AAS misuse only using logistic regression models because of small sample sizes for moderate and severe AAS misuse. We estimated the predicted probabilities of any AAS misuse for each level of GNC scores followed the regression models. In subsequent categorical analyses, we calculated sex-stratified adjusted odds ratios (AORs) with 95% CIs for the associations of GNC with three levels of AAS misuse, with gender conformity as the reference group. All statistical tests were considered to be statistically significant if two-tailed *P*-values < 0.05. Complete case analyses were used.

## Results

The weighted percentage (unweighted number) of students in the pooled sample was 49.67% (*n* = 9143) female, and 50.33% (*n* = 8611) male. Races/ethnicities were 15.44% (*n* = 2093) white, 25.80% (*n* = 3825) black, 48.24% (*n* = 8310) Hispanic/Latino, and 10.52% (*n* = 2936) other. Grade levels were 27.54% (*n* = 4540) in 9th grade, 25.80% (*n* = 4697) in 10th grade, 23.72% (*n* = 4400) in 11th grade, and 22.94% (*n* = 3921) in 12th grade. For sexual orientation, 83.45% (*n* = 14,342) of students reported heterosexual, 3.05% (*n* = 465) reported lesbian or gay, 8.55% (*n* = 1550) reported bisexual, and 4.95% (*n* = 825) were unsure about their sexual orientation.

The prevalence of GNC was 8.73%, and GNC varied by sex, race/ethnicity, and sexual orientation (Table 2). GNC was more prevalent among male students (12.85%) than female students (4.70%); was more prevalent among lesbian or gay (38.75%), bisexual (10.02%), and unsure (12.53%) students than heterosexual peers (7.06%); was more prevalent among black students (11.20%) than white (7.80%), Hispanic/Latino (7.20%), and other students (9.48%).

**Table 2:** Gender nonconformity among high school students by sex, race/ethnicity, grade, and sexual identity in selected US school districts, 2017–2019

Demographic group	Gender nonconformity, % (95% CI)		χ^2^*P*-value
	No	Yes	
Total population	91.27 (90.44–92.02)	8.73 (7.98–9.56)	NA
*Sex*			
Female	95.30 (94.61–95.91)	4.70 (4.09–5.39)	< 0.001
Male	87.15 (85.68–88.49)	12.85 (11.51–14.32)	
*Race/ethnicity*			
White	92.20 (90.04–93.93)	7.80 (6.07–9.96)	< 0.001
Black	88.80 (87.25–90.19)	11.20 (9.81–12.75)	
Hispanic/Latino	92.80 (91.82–93.67)	7.20 (6.33–8.18)	
All other races	90.52 (88.30–92.36)	9.48 (7.64–11.70)	
*Grade*			
9	89.91 (88.14–91.44)	10.09 (8.56–11.86)	0.074
10	91.44 (89.95–92.72)	8.56 (7.28–10.05)	
11	92.56 (91.04–93.85)	7.44 (6.15–8.96)	
12	91.71 (90.25–92.97)	8.29 (7.03–9.75)	
*Sexual orientation*			
Heterosexual	92.94 (92.10–93.70)	7.06 (6.30–7.90)	< 0.001
Lesbian/gay	61.25 (55.08–67.07)	38.75 (32.93–44.92)	
Bisexual	89.98 (87.34–92.12)	10.02 (7.88–12.66)	
Unsure	87.47 (83.18–90.78)	12.53 (9.22–16.82)	

CI, confidence interval; NA, not applicable

The prevalence of AAS misuse was 3.49% for any misuse, 1.17 for moderate misuse, and 0.47% for severe misuse. AAS misuse varied by sex and sexual orientation (Table 3). Male students reported higher prevalence of each of the three levels of AAS misuse than female students (any misuse: 4.66% vs. 2.33%; moderate misuse: 1.72% vs. 0.62%; and severe misuse: 0.68% vs. 0.27%). Any misuse was more prevalent among lesbian or gay (15.65%), bisexual (5.77%), and unsure (5.75%) students than heterosexual peers (2.44%). With a much lower prevalence, moderate misuse for sexual orientation shows a similar pattern to that of any misuse (Table 3).

**Table 3:** Non-prescription steroid use among high school students by sex, race/ethnicity, grade, and sexual orientation in selected US school districts, 2017–2019

Demographic group	Non-prescription steroid use, % (95% CI)		
	Any AAS misuse	Moderate AAS misuse	Severe AAS misuse
Total population	3.49 (3.03–4.02)	1.17 (0.93–1.46)	0.47 (0.35–0.63)
*Sex*			
Female	2.33 (1.89–2.86)***	0.62 (0.42–0.92)***	0.27 (0.14–0.51)*
Male	4.66 (4.01–5.42)	1.72 (1.37–2.14)	0.68 (0.46-1.00)
*Race/ethnicity*			
White	2.66 (1.73–4.07)	1.00 (0.50–2.01)	0.68 (0.29–1.56)
Black	3.97 (2.99–5.25)	1.50 (0.97–2.30)	0.54 (0.30–0.99)
Hispanic/Latino	3.55 (2.90–4.35)	0.99 (0.72–1.37)	0.43 (0.25–0.74)
All other races	2.14 (1.46–3.12)	0.56 (0.27–1.17)	0.06 (0.02–0.16)
*Grade*			
9	3.26 (2.51–4.22)	1.06 (0.66–1.70)	0.49 (0.26–0.92)
10	3.70 (2.95–4.64)	1.17 (0.84–1.63)	0.44 (0.27–0.72)
11	2.53 (1.96–3.26)	0.88 (0.60–1.28)	0.48 (0.26–0.89)
12	4.14 (3.06–5.58)	1.34 (0.89–2.03)	0.37 (0.18–0.76)
*Sexual orientation*			
Heterosexual	2.44 (2.09–2.85)***	0.82 (0.65–1.05)***	0.40 (0.28–0.56)
Lesbian/gay	15.65 (11.00-21.79)	3.61 (1.94–6.62)	0.89 (0.20–3.87)
Bisexual	5.77 (4.33–7.67)	1.79 (1.02–3.15)	0.65(0.30–1.42)
Unsure	5.75 (3.91–8.38)	2.70 (1.66–4.38)	1.09(0.47–2.54)

AAS, anabolic-androgenic steroids; CI, confidence interval

*P < 0.05; ***P < 0.001

In initial analyses, we estimated the prevalence of any AAS misuse for each GNC score (Table S2). Any misuse was not linearly associated with GNC score. After the multivariate logistic models, we estimated the predicted probabilities of any AAS misuse for each GNC score (Fig. S3). We can see that the probability of any AAS misuse was higher in most gender nonconforming female students (GNC score = 7), while was higher in moderate and most gender nonconforming male students (GNC score: 5–7).

In categorical analyses, among female students, GNC was associated with moderate and severe AAS misuse, but not with any AAS misuse. Moderate (AOR, 3.69; 95% CI 1.28–10.62) and severe (AOR, 5.00; 95% CI 1.05–23.76) AAS misuse were more likely among gender nonconforming students compared with gender conforming peers. Among male students, GNC was associated with AAS misuse at each of the three levels. Any (AOR, 4.75; 95% CI 2.93–7.69), moderate (AOR, 4.86; 95% CI 2.66–8.89) and severe (AOR, 4.13; 95% CI 1.43–11.95) AAS misuse were more likely among gender nonconforming students compared with gender conforming peers (Table 4).

**Table 4:** Sex-specific associations of non-prescription steroid use with gender nonconformity among high school students in selected US school districts, 2017–2019

Non-prescription steroid use	Gender nonconformity			
	No	Yes		
	%	%	OR (95% CI, *P*-value)	AOR (95% CI, *P*-value)
*Female students*				
Any AAS misuse	2.22	3.74	1.71 (0.90–3.23, *P* = 0.098)	0.85 (0.34–2.14, *P* = 0.734)
Moderate AAS misuse	0.59	1.57	2.70 (1.12–6.49, *P* = 0.027)	3.69 (1.28–10.62, *P* = 0.016)
Severe AAS misuse	0.25	0.92	3.70 (1.18–11.64, *P* = 0.025)	5.00 (1.05–23.76, *P* = 0.043)
*Male students*				
Any AAS misuse	2.88	17.23	7.03 (4.85–10.19, *P* < 0.001)	4.75 (2.93–7.69, *P* < 0.001)
Moderate AAS misuse	0.96	6.52	7.17 (4.28–12.02, *P* < 0.001)	4.86 (2.66–8.89, *P* < 0.001)
Severe AAS misuse	0.49	2.18	4.52 (1.82–11.23, *P* = 0.001)	4.13 (1.43–11.95, *P* = 0.009)

AAS, anabolic-androgenic steroids; OR, odds ratio (gender conformity is the referent group); CI, confidence interval; AOR, adjusted odds ratio (adjusted for race/ethnicity, grade, and sexual orientation, with gender conformity being the referent group)

## Discussion

This is the first study known to examine the association of GNC with lifetime AAS misuse independent of sexual orientation among high school students. The odds of lifetime AAS misuse significantly differed by gender expression, after adjusting for sexual orientation. Our findings provide the first epidemiological evidence of a unique link between GNC and lifetime AAS misuse.

In our study, near 9% of the students reported GNC, which is lower than the result of 3 large urban US school districts in 2017 YRBS [8]. The prevalence of GNC varied by demographic characteristics, including sex, race/ethnicity, and sexual orientation, which is consistent with previous research [8]. In particular, lesbian/gay students and bisexual students both reported higher prevalence of GNC than their heterosexual peers. However, GNC did not vary by grade in our study.

Despite great public interest in AAS misuse among adolescents, national YRBS data identified a declining linear trend in the prevalence of AAS misuse among US high school students during 2001–2017 (5.0–2.9%) [12]. In our study, the prevalence was 3.49% for any level of AAS misuse. Adolescent boys are at higher risk of exposure to AAS misuse at all levels than girls, and sexual minority adolescents are more likely to experience any and moderate levels of AAS misuse. The prevalence however did not vary by race/ethnicity or grade. Our findings echoed previous research demonstrating sexual orientation as a potential indicator of lifetime AAS misuse in adolescents [15, 21].

Our findings revealed that there were similarities in the association between GNC and AAS misuse among female and male students. Gender nonconforming female and male students both more likely to report AAS misuse at moderate or severe levels than gender conforming peers. According the minority stress model, increased social stress experienced by sexual minorities may explain at least partly the sexual orientation associated substance use [22], including AAS misuse [15]. From a social standpoint, the minority stress perspective suggests that at least some of the AAS misuse in sexual minorities occurs as a potential coping mechanism against social and minority stress [23]. Although GNC is associated with AAS misuse among both female and male students, the reasons behind the association may different. For gender nonconforming female students, using AAS would make them look more masculine as desired; while for gender nonconforming male students, AAS misuse may be a result of perceiving their bodies as inconsistent with current Western ideals for males (i.e., high muscularity) [15].

Association between GNC and AAS misuse also varied by sex. While the association between GNC and AAS misuse at any level was significant among male students, the prevalence of AAS misuse at any level did not vary by GNC among female students. One possible reason behind this sex difference is GNC is more acceptable for girls than for boys in Western social norms that masculine (competency) traits are valued more. For example, the term ‘sissy’ (gender nonconforming boy) is always used pejoratively, whilst ‘tomboy’ (gender nonconforming girl) is more acceptable now [24, 25]. Another possible reason is self-report bias. Gender nonconforming female students were no more likely to report AAS misuse than gender conforming female peers, which is due partly to the association between masculinity and a reticence to report AAS misuse [8]. Conversely, gender nonconforming male students may be more likely to disclose AAS misuse than gender conforming male peers partly because they are less inhibited by conventional masculinity norms [8].

We did not observe the dose-response association of GNC with AAS misuse, and are aware that no studies have examined the dose-response association. Researchers have found there is a linear relationship between GNC and other outcomes, including mental distress, suicidal thoughts, and bullying victimization [8, 20]. We did not have information on the motivations for AAS misuse because our data were derived solely from YRBS questionnaires, and YRBS did not measure it in any round of survey. The primary motivation for AAS misuse were physical appearance and muscular enhancement, and women were introduced to AAS commonly by a trusted man [26].

### Implications for clinical and educational practice

Given the potential numerous side effects associated with AAS misuse among adolescents, the possibility that an adolescent might misuse AAS as a result of GNC may be of interest to physicians and educators.

The AAS misusers are less likely to voluntarily disclose their usage to physicians [27]. In addition to physical dimensions, physicians working with adolescents should be observant of one’s GNC level and enquire gender identity concerns confidentially in identifying high risk individuals of AAS misuse [17]. Moreover, physician’s toolkit should include the knowledge of resources for psychological treatment, substance misuse intervention, and safety for gender nonconforming adolescents [8, 17]. Specifically, prevention programs on AAS misuse should be different from that of typical substance misuse. The motivation of AAS misuse (performance enhancement) is differ from that of typical substance misuse (pleasure effects associated with getting high). Their practices are also different. AAS is often misused as a long-term solution of physical effects, while typical substance is commonly used as a short-term coping mechanism for a variety of complex reasons [21].

School teachers have a duty of care towards all students, including gender nonconforming children and adolescents, under their supervision. The resource pack for schools should include the guidelines to help teachers without previous professional experience develop the confidence to manage the issue of GNC in their daily routines. Developing school-based programs that are inclusive of gender diversity in students may prevent substance misuse, including AAS misuse, and improve mental health in students by decreasing the stigma of GNC [8, 17]. Providing school teachers who are knowledgeable and supportive of GNC may also help to mitigate the GNC associated with stresses in the school environment [8].

### Limitations

Our study has some limitations. Of note, our study used a cross-sectional design, which can only provide an indication of association, not causality between GNC and AAS misuse. Furthermore, gender expression and AAS misuse can change over time, the YRBS data can not allow us to examine the effect of trajectories on the outcome of interest. Second, AAS misuse was measured with the lifetime use of steroids without a prescription in our study. Although AAS misuse is assessed over the lifetime, it may most likely occur relatively recently, because there is evidence that AAS misuse before 16 years is rare [13, 21]. Furthermore, AAS misuse in our study is vulnerable to overestimation bias because of possible ambiguity of the item. The wording of the item is “steroids” in YRBS, which does not explicitly state AAS [28], even though the item follows other items of illicit substances immediately [29]. Third, the binary GNC classification, based on the data distribution in our study, may not generalize directly to other studies with non-YRBS datasets. Fourth, the YRBS data only include participants who attend school, and sexual minority and gender minority youths may be less represented among high school students because they are more likely to drop out of school or do not attend school [30]. Fifth, our data are from selected US school districts, making our findings difficult to generalize to the US high school students in general.

## Conclusions

In this cross-sectional study using weighted district YRBS data, AAS misuse is associated with GNC. From a clinical perspective, it’s important for clinicians to be aware of gender expression when assessing risk factors for AAS misuse in routine adolescent health care. From an educational point of view, school environments that celebrate gender diversity are essential when developing adolescent health equity initiatives.

### Electronic supplementary material

Below is the link to the electronic supplementary material.

Supplementary Material 1.

## Data Availability

Data is available at https://www.cdc.gov/healthyyouth/data/yrbs/index.htm.
