# Supplementary material for: Nonconforming gender expression and adolescent anabolic-androgenic steroids misuse
Source: Child Adolesc Psychiatry Ment Health. 2024 Jun 6;18:65. doi: 10.1186/s13034-024-00761-9 (PMC11157812; doi:10.1186/s13034-024-00761-9)
Supplement: Supplementary file 1 — Supplementary Material 1. [file 13034_2024_761_MOESM1_ESM.docx]

S1 Table. Sample characteristics by sex in selected US school districts, 2017-2019

| **Variable** | **Unweighted no. (%)** | | **χ^2^ P-value** |
| --- | --- | --- | --- |
|  | **Female students** | **Male students** |  |
| **Demographic group** |  |  |  |
| Total population | 9,143(100) | 8,611(100) | NA |
| Race/ethnicity |  |  |  |
| White | 1,051(14.98) | 1,042(15.90) | 0.284 |
| Black | 2,102(26.73) | 1,723(24.86) |  |
| Hispanic/Latino | 4,277(48.21) | 4,033(48.27) |  |
| All other races | 1,478(10.08) | 1,458(10.97) |  |
| Grade |  |  |  |
| 9 | 2,313(26.96) | 2,227(28.12) | 0.715 |
| 10 | 2,506(25.63) | 2,191(25.97) |  |
| 11 | 2,269(24.06) | 2,131(23.38) |  |
| 12 | 1,974(23.35) | 1,947(22.53) |  |
| Sexual orientation |  |  |  |
| Heterosexual | 6,867(77.22) | 7,475(89.68) | <0.001 |
| Lesbian/gay | 233(2.86) | 232(3.24) |  |
| Bisexual | 1,271(13.60) | 279(3.49) |  |
| Unsure | 543(6.32) | 282(3.59) |  |
| **Non-prescription steroid use** |  |  |  |
| Any AAS misuse |  |  |  |
| No | 8,741(97.67) | 7,948(95.34) | <0.001 |
| Yes | 213(2.33) | 341(4.66) |  |
| Moderate AAS misuse |  |  |  |
| No | 8,901(99.38) | 8,167(98.28) | <0.001 |
| Yes | 53(0.62) | 122(1.72) |  |
| Severe AAS misuse |  |  |  |
| No | 8,936(99.73) | 8,243(99.32) | 0.026 |
| Yes | 18(0.27) | 46(0.47) |  |
| **Gender nonconformity** |  |  |  |
| No (score: 1-4) | 8,243(95.30) | 6,993(87.15) | <0.001 |
| Yes (score: 5-7) | 395(4.70) | 868(12.85) |  |

Abbreviation: AAS, Anabolic-androgenic steroids; NA, not applicable.

S2 Table. Sex-specific prevalence of non-prescription steroid use by gender nonconformity score in selected US school districts, 2017-2019

| **Non-prescription steroid use** | **Gender nonconformity score** | | | | | | |
| --- | --- | --- | --- | --- | --- | --- | --- |
|  | 1 | 2 | 3 | 4 | 5 | 6 | 7 |
| **Female students** |  | | | | | | |
| Any AAS misuse (%) | 2.88 | 1.92 | 2.60 | 1.55 | 2.27 | 4.10 | 7.34 |
| Moderate AAS misuse (%) | 0.71 | 0.55 | 0.63 | 0.45 | 0.91 | NA | 6.02 |
| Severe AAS misuse (%) | 0.30 | 0.22 | 0.28 | 0.22 | 0.39 | NA | 3.92 |
| **Male students** |  | | | | | | |
| Any AAS misuse (%) | 3.08 | 1.55 | 2.80 | 5.51 | 19.40 | 15.39 | 16.82 |
| Moderate AAS misuse (%) | 1.23 | 0.31 | 1.28 | 1.55 | 5.89 | 7.79 | 6.08 |
| Severe AAS misuse (%) | 0.92 | 0.11 | 0.50 | 0.43 | 0.88 | 2.71 | 2.86 |

Abbreviation: AAS, Anabolic-androgenic steroids; NA, not applicable (no observations).

S3 Figure. Predicted probabilities of gender nonconformity score for any steroid use. Predicted probabilities were calculated using Stata contrast and margins commands after the regression models adjusting for race/ethnicity, grade, and sexual orientation. Error bars indicate 95% CIs.
